# Associations between self-reported symptoms and circulating protein biomarkers: A scoping review protocol

**DOI:** 10.1371/journal.pone.0352015

**Published:** 2026-07-21

**Authors:** Marijane Luistro Jonsson, Tina Gustavell, Noora Sissala, Haris Babačić, Lars E. Eriksson

**Affiliations:** 1 Division of Nursing, Department of Neurobiology, Care Sciences and Society, Karolinska Institutet, Huddinge, Sweden; 2 Department of Upper Abdominal Diseases, Theme Cancer, Karolinska University Hospital, Stockholm, Sweden; 3 Department of Oncology-Pathology, Karolinska Institutet, Stockholm, Sweden; 4 Science for Life Laboratory (SciLifeLab), Stockholm, Sweden; 5 School of Health and Medical Sciences, City St George’s, University of London, London, United Kingdom; Universidade Estadual Paulista Julio de Mesquita Filho Faculdade de Medicina Campus de Botucatu, BRAZIL

## Abstract

**Background:**

Studies linking self-reported symptoms to circulating protein biomarkers are increasing, partly driven by the rising number of protein biomarkers being identified. Despite research advances, current reviews that synthesise these studies are typically disease-specific. A broader, diagnosis-agnostic and multidisciplinary scoping review can uncover shared biological patterns associated with symptoms across different conditions and can generate insights that advance precision health and symptom science. This scoping review protocol builds on this need for a broad approach as it aims to consolidate current knowledge on self-reported symptom and circulating protein biomarker associations, identify potential patterns, and provide relevant insights for clinical practice and future research.

**Methods:**

The protocol is aligned with the PRISMA and JBI guidelines for scoping reviews. The search strategy includes original peer-reviewed journal research articles examining associations between self-reported symptoms and protein biomarkers in blood plasma or serum. Searches will be conducted from PubMed, CINAHL, Embase, and Web of Science databases. A preliminary search retrieved more than 30,000 articles, prompting a pilot test of the Artificial Intelligence (AI)-assisted review screening tool ASReview. The pilot demonstrated time savings, refined methodological decisions, and confirmed the feasibility of proceeding with the review. Therefore, screening will be conducted using ASReview, with a total of five reviewers involved in the process. Data extraction will focus on single self-reported symptoms and circulating protein biomarker pairs that have a reported association. Analysis will involve counting and mapping these associations to identify potential patterns. The protocol was registered with the Open Science Framework (OSF) https://osf.io/bku3f.

**Discussion:**

This protocol provides a structured and transparent approach for conducting a large-scale scoping review. By adhering to established guidelines, the protocol provides a more comprehensive, standardised, exact, and reproducible accumulation of knowledge, while acknowledging potential limitations. The expected results can contribute to summarise the current understanding of associations between self-reported symptoms and circulating protein biomarkers. This fosters the integration of symptom science and the omics field, specifically proteomics, to advance precision health.

## Introduction

Precision health and symptom science are advancing healthcare by recognising how individual variability impacts health outcomes. On the one hand, precision health integrates an individual’s unique genetic, molecular, phenotypic, or imaging information with their lifestyle, behaviour, and environment [1–3]. On the other hand, symptom science explores the biological, psychological, and social mechanisms underlying the symptoms people experience [4,5]. These two fields converge through frameworks such as the Nursing Science Precision Health (NSPH) model. The NSHP model integrates precision health concepts that measure different levels of information with symptoms and self-management focusing on subjective experiences [6]. By combining these concepts into a single framework, measurable parameters can be integrated in relation to an individual’s experience.

In particular, the NSPH model depicts a cycle that begins with complex symptom measurement, phenotypic characterisation, biomarker identification, and eventually clinical application [7]. At each stage, precision health can be integrated through the use of supportive technology [8]. For instance, innovations in omics technologies, such as methods for systematic studies of genes (genomics), mRNAs (transcriptomics), proteins (proteomics), or metabolites (metabolomics), have accelerated biomarker discovery [9–12]. These advances enable comprehensive molecular analyses that provide knowledge to understand the links between complex biological processes and phenotypes [13–15]. As a result, they can serve as functional interfaces and mechanistic links between biological systems and self-reported symptoms.

Proteins are important to study because they directly capture the molecular changes that give rise to the presentation of symptoms [16–18]. While changes in genes and mRNA molecules can reflect predisposition to various conditions and be related to phenotypic changes, proteins reflect the real-time state of the body. Proteins are the final gene products and functional executors of biological processes. In addition, they are easily accessible and stable in readily available biofluids, and can be reliably measured with well-established methods. This gives proteins a high potential for translation into clinical applications. Therefore, proteomics can not only drive the discovery of biomarkers for a possible diagnosis [19–21], but also allow identifying correlations between proteome patterns and phenotypes across health and disease contexts [22,23]. As research in the proteomics field increasingly provides insight into the biological underpinnings of symptoms and disease progression, it can also complete the NSPH model cycle by potentially contributing to precision in clinical applications. Thus, this review focuses on proteins to maintain a manageable scope while identifying molecular patterns most relevant to symptom science and highlighting biomarkers with strong translational potential.

The disease-agnostic perspective adopted by the NSPH model allows the identification of common biological pathways across conditions, which can be crucial for unexplained, experienced symptoms that are often ignored and constrained by traditional diagnostic categories. Despite research advances, most studies examine the respective associations of symptoms or biomarkers with specific diseases [24–26]. However, there are some studies exploring associations between symptoms and biomarkers, but these are also restricted to specific disease [27–30]. In addition, the few reviews consolidating these studies typically concentrate on specific diseases [31] or symptoms [32], overlooking important associations already identified in other medical conditions. To address this need for a broader perspective, the proposed study takes a diagnosis-agnostic and multi-disciplinary approach to map existing evidence.

The research question for the review is “what is the current knowledge on the associations between self-reported symptoms and circulating protein biomarkers?”. The Population-Concept-Context (PCC) framework [33] can be used to structure the research question as follows: a) the population is adults (i.e., 18 years and older); b) the concept is the association between a single circulating protein biomarker (measured in blood plasma and serum) and a single self-reported symptom; and c) the context will consider all settings, regardless of any underlying syndrome, disease, or condition. The focus is on symptoms that are self-reported by patients to avoid filtering and observer biases of clinicians and other raters. It also looks at single symptoms and not composite measures because the latter can make it difficult to identify which symptom drives the association. The aggregation of multiple symptom domains can limit the interpretability of individual symptoms.

A scoping review, as opposed to other types of systematic review, is the most suitable way to address the broad research question, the goal of mapping existing evidence, and the large volume of fragmented and heterogeneous studies generated from the preliminary library database search [34–36]. Moreover, a search into PROSPERO, Inplasy, OSF, and PLOS One protocol registries confirmed that no existing review protocols address the same research question. Registered protocols on similar topics focus on specific diseases and symptoms [37,38], further justifying the study and distinguishing its novel and broader scope.

## Materials and methods

### Aim and research design

The aim of this scoping review is to consolidate current knowledge and identify potential patterns of self-reported symptoms and their association with circulating protein biomarkers. It also seeks to provide relevant insights that can inform clinical practice and guide future research.

The research design and methodology are based on the PRISMA guidelines for scoping reviews [39] and the JBI Scoping Review Methodology Group’s guide to conducting scoping reviews [40]. In addition, established principles are followed for conducting scoping reviews [34–36], systematic reviews of the literature [41–43], and review quality assessments [44]. A completed PRISMA-P checklist for review protocols [45,46] is provided in the Supporting Information (S3 Checklist) to ensure that the necessary items are addressed in this protocol. The protocol was registered with the Open Science Framework (OSF) https://osf.io/bku3f. As a scoping review, there is no patient and public involvement.

### Eligibility criteria

Table 1 presents the inclusion and exclusion criteria that will guide the selection of articles. With regard to symptoms, studies in which single self-reported symptoms are reported and measured will be included. This means that a distinct symptom has been identified, such as “shortness of breath”, “depressive mood” or “dyspnea”, and the patient has reported it. This excludes studies in which symptoms are grouped and reported only in the form of syndromes, composite symptoms scores, global indices, inventories, or other summarised symptom outcomes. However, studies that provide details on the association between a distinct symptom and a plasma protein, which are evaluated as part of the calculation of the summarised outcomes of the symptoms, will also be included. During the screening, a list of relevant and irrelevant scales will be developed and updated, which will help align the reviewers’ screening process.

**Table 1:** Inclusion and Exclusion Criteria.

Inclusion Criteria	Exclusion Criteria
Published, peer-reviewed studies, written in the English language, which involve adult human samples, AND Examines at least one association between a symptom and a circulating biomarker, AND \ The individual symptoms are self-reported, AND The circulating biomarkers are proteins, measured in blood plasma or serum	Reviews, protocols, conference abstracts, book chapters, books, comments, editorials, retractions, grey literature, pre-prints, and other publications not classified as peer-reviewed original journal publications, OR Studies involving pregnant women due to the population’s transient physiological characteristics that may not be applicable to the general adult population, OR Studies where symptoms are interpreted by a clinician, OR Studies where different and unrelated symptoms are combined, clustered, or aggregated to a single composite measure (e.g., “Quality of Life” measured as the combination of different functions and symptoms)

### Identification of information sources and search strategy

The search will involve the following bibliographic databases: PubMed, CINAHL, Embase, and Web of Science. The main concepts involved in the study are self-reported symptoms and circulating protein biomarkers, and the main keywords include symptoms, sensations, symptomology, quality of life, biomarker, plasma, serum, blood and circulating. S1 Appendix shows the exact search strategies to be used in the search of the various databases, which were developed and tested by the research team, and peer-reviewed by the library and literature review specialists at Karolinska Institute. The consolidation of records and the removal of duplicates will be done using Covidence [47] and EndNote [48], followed by manual checks for the remaining duplicates and exclusions that were not detected by the automation tools.

A preliminary search was conducted in September 2024, which yielded 34,606 articles that questioned whether the study was feasible. This led to a pilot study that investigated whether ASReview [49], an AI-assisted tool that sorts and ranks articles, can aid in the screening of articles. From testing a sample of 1005 articles, the results showed that ASReview can indeed streamline the screening process by identifying and prioritising articles relevant to the research question, urging adoption of the tool. The pilot study also helped refine the article selection criteria, the calibration process, and the methodological decisions. Details of the pilot study are discussed in the section on Pilot testing of screening process. An updated search will be performed after the pilot, as well as after the extraction process, to capture recent articles that were not included in the initial search.

### Data charting process

The title and abstract screening process will be charted within ASReview, using its real-time monitoring dashboards. Two reviewers will screen articles via a cloud version of ASReview, ensuring that the sequence of articles will always be updated. Progress will be monitored through density and recall metrics. Since calibration has been established among the reviewers during the pilot testing, they will continue with control and quality checks in the screening process to monitor the accuracy and consistency of the selection.

The stopping rule will be when at least 10% of the total articles have been screened and no relevant articles have been identified in screening the last 50 articles. This is consistent with the recommended practices when using ASReview [50] and is consistent with the results of the pilot test. When the stopping rule is met, additional measures will be employed to verify that relevant articles are not missing. An example is to use model-switching strategies, which have generally been shown to improve performance compared to using the default model alone [51]. With the model switching, the same stopping rule will be implemented, where screening will stop after 50 consecutive non-relevant articles have been identified. As an additional measure that relevant articles are not missed, we will randomly screen articles from the remaining unscreened pile until there are 50 consecutive non-relevant articles.

After the title and abstract screening, the selected articles will be entered in Covidence [47] for full-text screening and data extraction. Each full-text article will be read by two reviewers, relevant items will be extracted, and conflicts will be resolved by a third reviewer. To ensure prompt handling of disagreements, weekly meetings will be held within the team. In total, a pool of five reviewers will participate in the selection and extraction of full texts. Consistency between reviewers will be ensured through training procedures, as well as shared screening and extraction rules (e.g., common lists of terminologies, excluded symptom measurements, and biomarkers).

### Data items and expected outputs

The following information from the full text of each selected article will be manually extracted using Covidence [47]. Table 2 presents the items to be extracted and their operationalisation.

**Table 2:** Items and Operationalisation.

Extraction Items	Operationalisation Details
Title	Full and short
Authours	Lead authour/Last name
Country	LCountry in which the study was conducted
Year	Year article was published
Journal	Name of Journal
Aim	Aim of study
Setting	Hospital/clinic, community, primary care, other
Study Design	Design of Study
Symptoms	Specific individual, self-reported symptom (e.g., dyspnoea)
Symptom Assessment	Questionnaire/method/scales used to assess the symptom, and language of questionnaire
Biomarker	Specific individual biomarker (e.g., TNF-α)
Biomarker Sample Source	Serum or Plasma
Laboratory method	Method used to measure the biomarker (e.g., ELISA)
Statistical method	Method used for association analysis
Adjustments	Adjustments or covariates used in analysis
Association Description	Positive, negative, none
Disease (if applicable)	Name of disease and medication/treatment
N	Number of patients for specific association analysis
Effect size estimate	Effect size measures (e.g. Pearson’s r, Cohen’s d, Confidence Interval)
Time frame	Time between symptom assessment and blood collection
Population	Total size, demographics (age, sex, geography, relevant clinical characteristics and timeframe)

The primary and main outcome will be the identification of self-reported symptoms associated with specific circulating protein biomarkers. Lists of excluded symptom measurements (e.g., not self-reported symptoms) and biomarkers (i.e., not proteins) will be continuously updated for quick reference. Since retrieved articles are assumed to be heterogeneous, there will be a pre-defined list of terminology for self-reported symptoms and circulating protein biomarkers during data extraction that, if needed, will be updated to ensure consistency. Additional outcomes will cover information that can help to aggregate and map heterogeneous results (e.g., symptom classification). We will also include relevant numeric estimates of the association between self-reported symptoms and circulating protein biomarkers.

### Synthesis of results

The results will be synthesised using the PRISMA flow diagram [52,53], showing an overview of the identification, screening, and inclusion results. For selected articles, we will record each time an association between symptom and protein is reported, regardless of whether the association was significant or not. Furthermore, symptom-biomarker associations will be analysed by looking at the counts, direction of the associations, and the strength of the association determined through statistical estimates. We will also explore patterns of re-categorising, aggregating, and grouping self-reported symptoms that are closely connected to each other (e.g., breathing symptoms) and present the results visually as graphs or matrices, and network analyses. The summarisation of results can reveal interesting patterns and clusters, which will need more refined analyses that future studies can explore.

### Data Management

Excel files will be used to manage data from the screening and extraction stages. It will also be the format used for data cleaning and exploratory analyses. These data files will be stored on the Karolinska Institute server and will be archived in different versions to securely track changes.

Since the study does not involve the recruitment of participants and will not require access to medical records or archived samples of individuals, ethical approval is not required and obtaining participant consents is not relevant. The study only deals with the final results of published articles, which are aggregated and anonymous. The registration of this protocol improves the transparency of the study, particularly the measures used to evaluate the sources of evidence. The findings will be disseminated through seminars and conference presentations, as well as publications.

We will make all materials related to this review available in a public repository after publication, including: (a) complete search strategies, (b) screening decisions, and (c) extracted datasets of symptom–biomarker associations. All analytical scripts (R and Python code used for ASReview, data processing, and visualisation) will be deposited on GitHub and archived via Zenodo with a DOI.

### Timeline

The proposed study is ongoing, with record screening in progress, and final results are expected in 2026. Table 3 gives an estimated project timeline:

**Table 3:** Timeline.

Stages	Estimated Schedule	Status
Preliminary Search	September 2024	Completed
Project Planning and Pilot Test	October-December 2024	Completed
Protocol Writing and Submission	January-February 2025	Completed
Record Screening	Expected to be done by late Spring 2026	Ongoing
Updated Search, Data Collection and Extraction	Expected to be done by Summer/Autumn 2026	To be done
Analysis of Results	Expected to be done by Autumn 2026	To be done
Manuscript Writing and Submission	Expected to be done by end of 2026	To be done

### Pilot testing of screening process

A preliminary search, covering articles from January 1985 to August 2024, retrieved 34,606 articles. After removing duplicates and those that meet the exclusion criteria, there were still 20,412 articles. This led to the testing of whether ASReview could streamline the screening process. Previous studies have shown that using ASReview reduces the number of publications that need to be screened while still finding relevant articles, in contrast to manual random screening, which is error prone and time consuming [54,55]. To start with, the software needs prior knowledge (i.e., a set of articles marked as relevant and irrelevant) to identify relevant articles within a pile of articles that are to be screened. The software ranks the relevance of the articles in the pile based on this prior knowledge and then continuously adjusts the ranks based on the reviewer’s decision whether the article in the pile is relevant or irrelevant. Screening stops when non-relevant articles start appearing sequentially, based on a pre-defined stopping rule.

The pilot sample involved 1005 records from the pile (1000 from Web of Science to be screened, and 5 manually included as prior knowledge). Using ASReview, two reviewers sequentially screened the pilot sample, based on titles and abstracts (i.e., one reviewer started by assessing the relevance and the other double checked if they agreed with the decision). In cross-checking the results, articles with contrasting assessments were jointly discussed, and the remaining disagreements were resolved by discussion and consensus with other team members. The two reviewers independently screened 1,000 records with 110 disagreements. This results in substantial inter-rater reliability (Cohen’s κ=0.77, 95% CI 0.74–0.80), indicating strong agreement beyond chance. This is part of the work to calibrate the selection of reviewers. The pilot yielded 265 relevant articles (26%), later refined to 104 (10%) after manual assessment of the full texts.

The process behind conducting the pilot test was guided by the procedures and heuristics used by other studies, which often suggests screening a random set [50,56–58]). In general, the pilot test helped refine the selection criteria of the articles, aid in the calibration process, and assist our methodological decisions. For example, it fostered our decision to deploy a cloud-based version of ASReview to allow multiple concurrent accesses and to allow independent screening among reviewers. Performance metrics from the pilot, generated by simulating the different AI models (i.e., machine learning), also confirmed that ASReview improves screening efficiency and indicated that the different AI models within ASReview performed similarly, allowing flexibility in model selection (S2 Appendix). Specifically, performance metrics which are described more in S2 Appendix included recall, work saved over sampling, additional relevant records found, and average time to discover. The results support the feasibility of the study and led to the decision to proceed with ASReview’s default AI model to screen the remaining large volume of articles. The output of the pilot test (i.e., relevant and non-relevant articles) will be used as the prior knowledge for the larger dataset. The pilot data are available on the GitHub repository https://github.com/larseriksson1group/Scoping-Review-Protocol.

## Discussion

This study protocol outlines the research team’s approach to conducting a scoping review on the associations between self-reported symptoms and circulating protein biomarkers, and incorporates insights from a pilot study that used an AI-assisted tool to screen a large data set. The pilot study provided valuable support in refinement of methodological decisions, which are detailed in this protocol. By elaborating on the plans for the review process, the protocol ensures methodological transparency and aligns with established guidelines.

Potential limitations and challenges in conducting the full scoping review include the large number of heterogeneous articles, which can slow down the extraction process and analysis. Differences in reporting practices, the use of concepts, and study designs across different disciplines can require additional terminology adjustments and calibration. Major protocol deviations will be documented and reported.

Several possible sources of bias can be anticipated in this scoping review, such as language-of-publication bias, publication bias, and technical bias due to heterogeneity in proteomics methods. Although language-of-publication bias could be mitigated by removing language inclusion restrictions, including non-English manuscripts would require additional translation resources and entail risk of translation-induced bias. We deemed this bias acceptable since most of the published articles are in English and our preliminary searches have already identified a large number of articles.

Furthermore, we acknowledge that heterogeneity in biomarker assays can limit comparability between studies. We will mitigate this issue by noting the type of laboratory method used, be transparent with the degree of heterogeneity, and discuss how this affects our conclusions as well.

In conclusion, the expected results of this scoping review can contribute to the scientific community by consolidating the knowledge about associations between self-reported symptoms and circulating protein biomarkers. This can generate hypotheses for further investigation in other studies. The findings can also provide insights and implications for clinical practice by broadening the understanding of these associations in various conditions through a diagnosis-agnostic approach. Finally, the study can potentially advance precision health by bridging the fields of symptom science and proteomics and fostering the integration of individual circulating protein biomarkers into personalised care.

## Supporting information

S1 Appendix Search Strategy. This file contains details of the search strategies used in searching the different databases.
(PDF)

S2 Appendix Pilot test analyses. This file contains the analyses for the pilot screening, particularly the recall results, simulations of the different ASReview models, and comparison of the performance metrics of the different ASReview models.
(PDF)

S3 Checklist PRISMA-P Checklist. This file contains a completed PRISMA-P checklist, indicating how the necessary items are addressed in this protocol.
(PDF)
