# Supplementary material for: Associations between self-reported symptoms and circulating protein biomarkers: A scoping review protocol
Source: PLoS One. 2026 Jul 21;21(7):e0352015. doi: 10.1371/journal.pone.0352015 (PMC13387579; doi:10.1371/journal.pone.0352015)
Supplement: S1 Appendix — This file contains details of the search strategies used in searching the different databases. (PDF) [file pone.0352015.s001.pdf]

S1 Appendix: Search Strategy

Table 1. Search strategy

**PubMed**	((“symptoms”[Title/Abstract] OR “sensations”[Title/Abstract] OR “signs”[Title/Abstract] OR “Symptomology”[Title/Abstract] OR “quality of life”[Title/Abstract]) AND ((“biomarkers”[Title/Abstract] OR “biomarkers”[MeSH Terms:noexp]) AND (“plasma”[Title/Abstract] OR “plasma”[MeSH Terms:noexp] OR “blood”[Title/Abstract] OR “blood”[MeSH Terms:noexp] OR “serum”[Title/Abstract] OR “serum”[MeSH Terms:noexp] OR “circulating”[Title/Abstract])) NOT (“animals”[MeSH Terms:noexp] OR “animals”[Title/Abstract]))
**Cinahl**	TI ((Symptoms OR Sensations OR Signs OR Symptomology OR ”Quality of Life”) AND (Biomarker AND (plasma OR blood OR serum OR circulating)) NOT animals) OR AB ((Symptoms OR Sensations OR Signs OR Symptomology OR ”Quality of Life”) AND (Biomarker AND (plasma OR blood OR serum OR circulating)) NOT animals)
**Embase**	(symptoms:ti,ab,kw OR sensations:ti,ab,kw OR signs:ti,ab,kw OR symptomology:ti,ab,kw OR ’quality of life’:ti,ab,kw) AND biomarker:ti,ab,kw AND (plasma:ti,ab,kw OR blood:ti,ab,kw OR serum:ti,ab,kw OR circulating:ti,ab,kw) NOT animals:ti,ab,kw
**Web of Science**	TS=((Symptoms OR Sensations OR Signs OR Symptomology OR ”Quality of Life”) AND (Biomarker AND (plasma OR blood OR serum OR circulating)) NOT (Animals))
