# Supplementary material for: Associations between self-reported symptoms and circulating protein biomarkers: A scoping review protocol
Source: PLoS One. 2026 Jul 21;21(7):e0352015. doi: 10.1371/journal.pone.0352015 (PMC13387579; doi:10.1371/journal.pone.0352015)
Supplement: S2 Appendix — This file contains the analyses for the pilot screening, particularly the recall results, simulations of the different ASReview models, and comparison of the performance metrics of the different ASReview models. (PDF) [file pone.0352015.s002.pdf]

S2 Appendix: Pilot study analyses

Based on available metrics and our analyses of the pilot test, we found that ASReview accelerated the identification of relevant articles compared to random screening (see Fig 1). ASReview simulation capabilities allowed us to compare different AI models, and we also found that they performed similarly with our pilot data, indicating flexibility in model selection for scaling up (Fig 2).

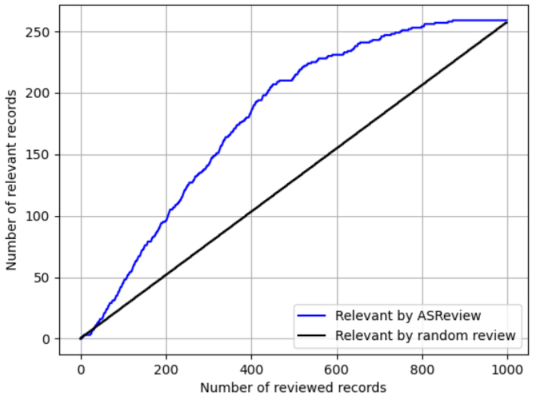

Fig 1. Recall Results. Number of relevant records found over number of reviewed records during screening using ASReview (blue) vs random screening (black).

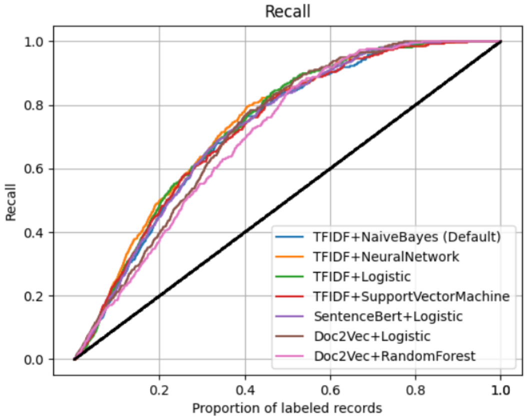

Fig 2. Results of simulations in comparing different ASReview models. Classifier parameters are the following: TF-IDF (Term Frequency-Inverse Document Frequency) measures how often and rare a term appears; Naïve Bayes assumes that the presence of a particular feature in a class is unrelated to the presence of any other feature; Logistics estimates the probability that a given input point belongs to a certain class; SVM (Support vector machine) finds the optimal hyperplane or decision boundary that best separates data points of different classes; NN (Neural Network) recognizes patterns and learns complex relationships in data; SBert (Sentence-BERT) computes sentence embeddings; Doc2Vec are neural network-based approach that learns the distributed representation of documents.

Regarding performance metrics (i.e., worked saved over sampling, extra relevant records found, average time to discover), our analysis did not identify a particular model that significantly outperformed others (Table 1). The best performers were not significantly different from others. These findings and insights from the pilot test guided our methodological decision to pursue using ASReview with its default model in screening the remaining large volume of articles, to make the project more efficient to conduct.

Table 1. Comparison of performance metrics among different AI models in ASReview

**Model**	**WSS@.95**	**ERF@.10**	**ATD**
TF-IDF+NaiveBayes	0.2633	0.1274	275.6139
TF-IDF+RandomForest	0.2923	0.0656	285.1622
TF-IDF+Logistic	0.3083	0.1429	263.8880
TF-IDF+SVM	0.2683	0.1467	273.2857
TF-IDF+NN	0.3013	0.1544[2]	260.4401[3]
SBert+RandomForest	0.1942	0.1081	285.8764
SBert+Logistic	0.2883	0.1236	271.8687
SBert+SVM	0.3003	0.1120	288.6216
SBert+NN	0.0310	0.1274	305.9537
Doc2Vec+RandomForest	0.3083	0.0888	294.7799
Doc2Vec+Logistic	0.3243[1]	0.1120	277.0039
Doc2Vec+SVM	0.3013	0.0733	297.3745
Doc2Vec+NN	0.0290	0.0849	360.3552

[1]Best performance in WSS@.95
[2]Best performance in ERF@.10
[3]Best performance in ATD
WSS@.95 (Work Saved over Sampling at 95% Recall): (Work Saved over Sampling at 95% Recall): proportion of relevant records screener does not have to screen at recall of 95% of the total records
ERF@.10 (Extra Relevant records Found at 10% Recall): proportion of relevant records screener found compared to the number of relevant records found via random screening at recall of 10%
ATD (Average Time to Discover): indicates how many records screener needs to be screen on average to find all relevant records in the dataset
