# Supplementary material for: Associations between self-reported symptoms and circulating protein biomarkers: A scoping review protocol
Source: PLoS One. 2026 Jul 21;21(7):e0352015. doi: 10.1371/journal.pone.0352015 (PMC13387579; doi:10.1371/journal.pone.0352015)
Supplement: S3 Checklist — This file contains a completed PRISMA-P checklist, indicating how the necessary items are addressed in this protocol. (PDF) [file pone.0352015.s003.pdf]

PRISMA-P 2015 Checklist

Protocol Title: "Associations between self-reported symptoms and circulating protein biomarkers: A scoping review protocol"

Section/topic	#	Checklist item	Information reported: Yes	Information reported: No	Line number(s)
**ADMINISTRATIVE INFORMATION**					
**Title**					
Identification	1a	Identify the report as a protocol of a systematic review	x	☐	p.1, Title (protocol for scoping instead of systematic review)
Update	1b	If the protocol is for an update of a previous systematic review, identify as such	☐	x	N/A
**Registration**	2	If registered, provide the name of the registry (e.g., PROSPERO) and registration number in the Abstract	x	☐	lines 23-24, p. 2
**Authors**					
Contact	3a	Provide name, institutional affiliation, and e-mail address of all protocol authors; provide physical mailing address of corresponding author	x	☐	p.1
Contributions	3b	Describe contributions of protocol authors and identify the guarantor of the review	x	☐	Entered in the submission system.
**Amendments**	4	If the protocol represents an amendment of a previously completed or published protocol, identify as such and list changes; otherwise, state plan for documenting important protocol amendments	☐	x	N/A
**Support**					
Sources	5a	Indicate sources of financial or other support for the review	x	☐	Entered in the submission system.
Sponsor	5b	Provide name for the review funder and/or sponsor	x	☐	Entered in the submission system.
Role of sponsor/funder	5c	Describe roles of funder(s), sponsor(s), and/or institution(s), if any, in developing the protocol	☐	x	N/A
**INTRODUCTION**					
**Rationale**	6	Describe the rationale for the review in the context of what is already known	x	☐	34-116, pp.3-4 (Introduction; Aim and research design)

Section/topic	#	Checklist item	Information reported Yes	No	Line number(s)
**Objectives**	7	Provide an explicit statement of the question(s) the review will address with reference to participants, interventions, comparators, and outcomes (PICO)	x	☐	82-93, pp- 3-4<br>(Introduction)<br>(PCC instead of PICO)
**METHODS**					
**Eligibility criteria**	8	Specify the study characteristics (e.g., PICO, study design, setting, time frame) and report characteristics (e.g., years considered, language, publication status) to be used as criteria for eligibility for the review	x	☐	103-116, p. 4<br>(Aim and Research Design)<br><br>118-128, p. 4<br>Table 1, p.5<br>(Eligibility Criteria: - Inclusion/Exclusion Criteria)
**Information sources**	9	Describe all intended information sources (e.g., electronic databases, contact with study authors, trial registers, or other grey literature sources) with planned dates of coverage	x	☐	129-150, p.5<br>(Identification of sources and search strategy)<br><br>224-265, pp.8-9<br>(Pilot testing of screening process)
**Search strategy**	10	Present draft of search strategy to be used for at least one electronic database, including planned limits, such that it could be repeated	x	☐	S1 Appendix<br><br>129-150, p.5<br>(Identification of sources and search strategy)
**STUDY RECORDS**					
Data management	11a	Describe the mechanism(s) that will be used to manage records and data throughout the review	x	☐	204-220, p.7<br>(Data management)<br><br>221-223, p.7<br>Table 3, p. 8<br>(Timeline)
Selection process	11b	State the process that will be used for selecting studies (e.g., two independent reviewers) through each phase of the review (i.e., screening, eligibility, and inclusion in meta-analysis)	x	☐	151-177, p.5-6<br>(Data charting process)

Section/topic	#	Checklist item	Information reported Yes	Information reported No	Line number(s)
					224-265, pp. 8-9<br>(Pilot testing of screening process)<br><br>S2 Appendix
Data collection process	11c	Describe planned method of extracting data from reports (e.g., piloting forms, done independently, in duplicate), any processes for obtaining and confirming data from investigators	x	☐	178-191, p. 6<br>(Data items and expected outputs)
**Data items**	12	List and define all variables for which data will be sought (e.g., PICO items, funding sources), any pre-planned data assumptions and simplifications	x	☐	Table 2, p.7
**Outcomes and prioritization**	13	List and define all outcomes for which data will be sought, including prioritization of main and additional outcomes, with rationale	x	☐	178-191, p.6<br>(Data items and expected outputs)
**Risk of bias in individual studies**	14	Describe anticipated methods for assessing risk of bias of individual studies, including whether this will be done at the outcome or study level, or both; state how this information will be used in data synthesis	☐	x	N/A
**DATA**					
**Synthesis**	15a	Describe criteria under which study data will be quantitatively synthesized	☐	x	N/A
	15b	If data are appropriate for quantitative synthesis, describe planned summary measures, methods of handling data, and methods of combining data from studies, including any planned exploration of consistency (e.g., I^2, Kendall's tau)	☐	x	N/A
	15c	Describe any proposed additional analyses (e.g., sensitivity or subgroup analyses, meta-regression)	☐	x	N/A
	15d	If quantitative synthesis is not appropriate, describe the type of summary planned	x	☐	192-203, p.6<br>(Synthesis of Results)
**Meta-bias(es)**	16	Specify any planned assessment of meta-bias(es) (e.g., publication bias across studies, selective reporting within studies)	x	☐	266-298, p.9<br>(Discussion)
**Confidence in cumulative evidence**	17	Describe how the strength of the body of evidence will be assessed (e.g., GRADE)	☐	x	N/A

This checklist has been adapted for use with protocol submissions to *Systematic Reviews* from Table 3 in Moher D et al: Preferred reporting items for systematic review and meta-analysis protocols (PRISMA-P) 2015 statement. *Systematic Reviews* 2015 **4**:1
